# Supplementary material for: Development of the Menu Assessment Scoring Tool (MAST) to Assess the Nutritional Quality of Food Service Menus
Source: Int J Environ Res Public Health. 2023 Feb 23;20(5):3998. doi: 10.3390/ijerph20053998 (PMC10001456; doi:10.3390/ijerph20053998)
Supplement: Supplementary file 1 [file ijerph-20-03998-s001.zip › Supplementary Table S1.pdf]

Supplementary Table S1: Food business classification framework

**Food retail**	**Food service**	**Charitable food provision**	**Food production and preparation**	**Institutional food**	**Accommodation and recreation services**
Supermarket<br>Discount grocery store<br>Other grocery store<br>Convenience store<br>World food/ethnic food store<br>Health food store<br>Butcher/poultry store<br>Fishmonger<br>Bakery<br>Fruit and vegetable store/greengrocer<br>Other specialist food retail outlet<br>Liquor merchant/bottle shop<br>General retail, not food or liquor	Café/coffee shop<br>Restaurant<br>Fast casual/quick service/takeaway<br>Pub/tavern/bar/winery/distillery	Emergency food provision - groceries<br>Emergency food provision - meals<br>Meals-on-wheels	Catering kitchens/home-based catering business/cooking classes<br>Food truck/coffee or other drinks van/food market vendor<br>Food home delivery service<br>Food manufacturer/processor<br>Packer, warehouse, food storage, food producer, wholesaler	Hospitals<br>Residential care<br>Defence<br>Correctional<br>Corporate (workplace)<br>Education<br>Childcare<br>Community centre/church/hall/ function centre<br>Residential worksite<br>Other	Entertainment venue<br>Health and leisure venue<br>Member based clubs<br>Accommodation with food
